# Knowledge, attitudes, and beliefs about HIV pre-exposure prophylaxis among US Air Force Health Care Providers

**DOI:** 10.1097/MD.0000000000004511

**Published:** 2016-08-12

**Authors:** Shilpa Hakre, Jason M Blaylock, Peter Dawson, Charmagne Beckett, Eric C Garges, Nelson L Michael, Patrick J Danaher, Paul T Scott, Jason F Okulicz

**Affiliations:** a US Military HIV Research Program, Walter Reed Army Institute of Research, Silver Spring, MD; b Henry M. Jackson Foundation for the Advancement of Military Medicine; c Walter Reed National Military Medical Center, Bethesda, MD; d The EMMES Corporation, Rockville, MD; e Navy Bloodborne Infection Management Center, Bethesda, MD; f Army Public Health Center (Provisional), Aberdeen Proving Ground, MD; g Infectious Disease Service, San Antonio Military Medical Center, Fort Sam Houston, San Antonio, TX.

**Keywords:** HIV pre-exposure prophylaxis, US Air Force, providers

## Abstract

Providers are central to effective implementation of HIV pre-exposure prophylaxis (PrEP). Primary care providers (PCP) and infectious disease physicians (ID) in the US Air Force (USAF) participated in a cross-sectional survey regarding knowledge, attitudes, and beliefs toward HIV PrEP. Characteristics associated with PrEP knowledge were assessed in univariate and multivariate analyses.

Among 403 (40% of 1015 providers) participants, 9% (PCP 383, ID 20) ever prescribed PrEP. In univariate analysis, years in practice, number of HIV-infected patients treated in the past 12 months, past prescription of antiretrovirals for HIV prevention, frequency of prescribing PrEP in the past 12 months, and ever being questioned by a patient about PrEP were associated with PrEP knowledge (*P* < 0.05). In multivariate analysis, providers who had ever prescribed antiretrovirals to prevent HIV (AOR: 2.37, 95% CI: 1.27–4.42) had greater odds of high PrEP knowledge. Despite concerns about medication side effects (overall 67%: PCP 68%, ID 85%) and prescribing PrEP without clear evidence (overall 60%: PCP 65%, ID 62%), 64% (PCP 65%, ID 85%) of participants indicated PrEP should be offered in the Military Health System and 68% (PCP 70%, ID 100%) disagreed with the statement that their patient population was not at risk for HIV infection.

Successful PrEP implementation in the USAF will require continued education and training of primary care providers to improve knowledge and mitigate concerns about PrEP.

## Abstract 2

Supplemental Digital Content is available in the text

## 1 Introduction

Following safety and efficacy evidence from clinical trials,[ ^1^ ^2^] the United States (US) Food and Drug Administration (FDA), on July 16, 2012, announced its approval of Truvada, an oral, fixed-dose combination of the antiretroviral drugs tenofovir disoproxil fumarate (TDF) and emtricitabine (FTC), for use in conjunction with a Risk Evaluation and Mitigation Strategy (REMS, primarily a training and education program for prescribers) to prevent sexual transmission of HIV among uninfected individuals at high risk of infection. ^[3]^ Since January 2011, when the Centers for Disease Control and Prevention (CDC) first issued guidance on the use of Truvada for pre-exposure prophylaxis (PrEP) among men who have sex with men (MSM), the CDC has provided updated guidance on PrEP use for heterosexual adults (August 10, 2012), ^[4]^ injection drug users (June 12, 2013) ^[5]^ and most recently, clinical practice guidelines for health care providers (May 2014). ^[6]^ Despite FDA approval of Truvada and updated CDC guidance on PrEP, studies conducted in the US indicate under-utilization of PrEP by health care providers, although this is improving. [^7^ ^8^ ^9^] Barriers to utilization include concerns about costs, viral resistance, patient adherence, and medication side effects.[ ^10^ ^11^]

Although active duty US Air Force (USAF) members with HIV currently total ∼250 cases and the new diagnosis rate of HIV among active duty personnel has been stable from 2010 through 2014 (0.14–0.17 per 1000 tested), ^[12]^ epidemiologic analyses of more than a decade of data (1996–2011) indicated risk for HIV acquisition was not evenly distributed and certain subgroups were at higher risk of infection. ^[13]^ Following the repeal of the US military's Don’t Ask Don’t Tell policy in 2011, data suggest MSM in the USAF are at highest risk for HIV infection, mirroring US national data. Among 316 HIV-infected active duty USAF personnel screened for gonorrhea and *Chlamydia trachomatis* from January 2010 through May 2014, 79% reported same sex sexual contact (71% MSM and 8% bisexual men vs 18% heterosexual men and women). ^[14]^ In the US, 78% of new HIV infections among men were attributable to same sex contact and an estimated 25% of HIV-uninfected MSM aged 18 to 59 years were eligible for PrEP from 2007–2012 according to 2014 CDC clinical guidelines.[ ^15^ ^16^] Although the USAF has HIV prevention strategies in place, such as random drug testing, biennial interval force HIV testing, and treatment-as-prevention with HIV-infected individuals initiating antiretroviral therapy (ART) early after HIV diagnosis, the uptake of PrEP by patients and providers is unknown especially in the absence of a USAF-specific PrEP clinical practice guideline. To inform future implementation of PrEP as a complement to current HIV prevention strategies in the USAF, we surveyed active duty (i.e., in active service) primary care providers (PCP) and infectious disease physicians (ID) to ascertain PrEP knowledge, attitudes, and beliefs.

## 2 Methods

### 2.1 Study population

On December 7, 2015, the director of the Air Force HIV Medical Evaluation Unit (San Antonio, TX) contacted active duty primary care providers (physicians, physician assistants, and nurse practitioners) and infectious disease physicians by email with an invitation to participate in a web-based needs assessment survey. The survey link was available for a 2-week time period in December 2015 and nonresponders received up to 3 reminder emails. Respondents were offered a $10 Amazon card as compensation for their time. Providers eligible for participation were ascertained from the Air Force Personnel Center (Randolph Air Force Base, TX) based on current occupation codes.

The needs assessment survey was conducted as a part of an HIV/bloodborne pathogen threat reduction project that was reviewed and approved as a public health activity and not human subject research by the Army Public Health Center's (Provisional, formerly US Army Public Health Command) Public Health Research Board (#14–311), and the Walter Reed Army Institute of Research's Institutional Review Board (#1861E).

### 2.2 Survey

The survey consisted of 20 multiple choice and short answer questions, of which a majority were adapted from surveys reported in the literature. [^17^ ^18^ ^19^ ^20^ ^21^] Providers were asked about demographics (3 questions), overall medical practice and PrEP experience (6 questions), attitudes (5 questions), and knowledge (6 questions) regarding PrEP.

### 2.3 Data management and analysis

Participants’ demographic, medical practice and PrEP experience, attitudes toward and knowledge of PrEP were described, overall, and by ID or noninfectious disease (non-ID) specialty which included family medicine, flight medicine, internal medicine, or other primary care specialties. The relationship of demographic and medical practice characteristics with knowledge scores was evaluated in univariate analysis using univariate logistic regression. Multivariate logistic regression was used to assess characteristics independently associated with high knowledge scores by adjusting for other variables of significance (*P* < 0.25).

Self-reported current state of medical practice was categorized into 5 regions: northeast, midwest, southern, western US, and outside the continental United States (OCONUS). Overall past experience of prescribing PrEP was derived from a question on past practice of prescribing antiretroviral medications for HIV post-exposure prophylaxis (PEP), nonoccupational PEP (nPEP), or PrEP. Using the median score as cutoff, PrEP knowledge was categorized into high (≥7 points) or low (0–6 points) knowledge. Points were based on scoring responses to 6 questions for a possible total score of 10 points. Five of 6 knowledge-based questions, adapted from Blumenthal et al, pertained to results of efficacy from PrEP clinical trials, (1) the iPrEX trial, (2) the Partners PrEP trial, (3) the VOICE and FEM PrEP trials, and (4) the drug combination approved by the FDA for PrEP, and (5) timeframe for monitoring medication side effects and laboratory toxicities. ^[17]^ The last question consisted of 5 responses, each worth 1 point, and referred to CDC guidance regarding risk assessment and clinical eligibility for initiating PrEP such as a negative HIV antibody test, symptoms and testing for acute HIV infection, confirmation of risk, and screening for hepatitis B and sexually transmitted infections. ^[6]^ Internal consistency of the 10 items used to evaluate knowledge was assessed with Cronbach's standardized alpha coefficient. ^[22]^ Data management and analysis were conducted using Statistical Analysis Software version 9.4 (SAS Cary, NC).

## 3 Results

### 3.1 Provider background and experience

Among 1172 eligible providers, 1015 were established as having received an invite; 157 invites were undeliverable either due to a full mailbox or an expired email address. Among invitees, 404 (40%) providers participated including 20/20 ID, 383/995 non-ID, and 1 registered nurse (excluded from analysis as a nonprescriber) resulting in an effective sample size of 403 providers.

**Figure 1:**
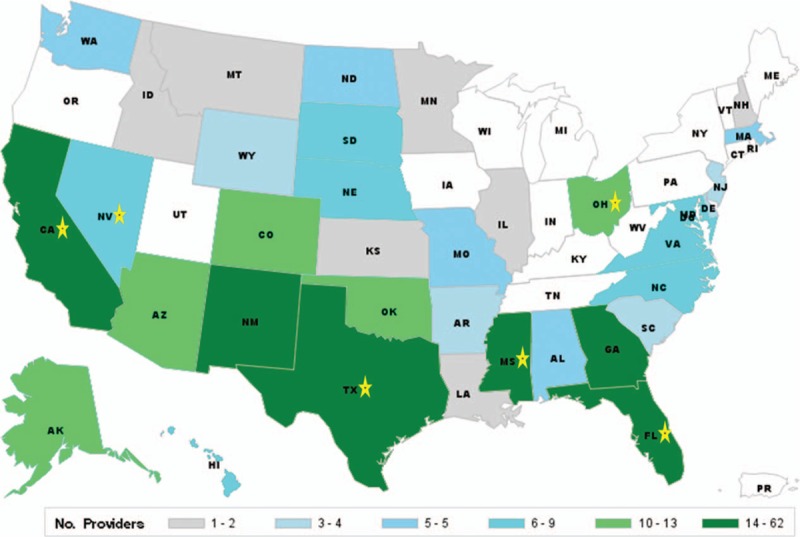
Geographic distribution of 403 participants’ medical practices. Overseas locations of practice are not included in the figure for 63 providers; these were in Germany, Italy, Estonia, Saudi Arabia, United Arab Emirates, Turkey, Afghanistan, Japan, Korea, and Guam. A star illustrates practice locations for all infectious disease providers in the United States and, overseas, in Germany.

Participants were a median age of 35.0 years (interquartile range [IQR] 31.5–41.0), more than half (59%) were male, and a majority (74%) reported being of white non-Hispanic racial ethnic origin (Table 1). A majority of participants were trained in family medicine (58%) and were physicians (64%). Respondents were representative of eligible providers who were primarily physicians (64%), of family medicine specialty (57%), and located in southern US (42%) (Table 1). Participants were licensed providers in the US for a median of 5.0 years (IQR 3.0–9.0), primarily practiced in southern US states (46%) (Fig. 1), and reported a median patient volume of 200.0 per month (IQR 80.0–360.0). The majority of providers rated their knowledge of PrEP as poor (overall 59%: ID 5%, non-ID 62%) and had never prescribed PrEP or PEP (overall 72%: ID 0%, non-ID 76%) (Table 1, Supplemental Table 1). Overall 26% reported having prescribed antiretroviral therapy to prevent HIV infection, commonly (21%) for occupational PEP. Only 9% of providers (75% of ID, 5% of non-ID) reported ever prescribing PrEP (Table 1) with prescriptions being infrequent on a monthly basis over the 12 months before the survey (median prescriptions, IQR: overall 0.2, 0.0–6.0; ID 2.0, 0.0–3.5; non-ID 0.0, 0.0–1.0). Only 38% of respondents (ID 95%, non-ID 34%) reported ever being questioned by a patient about PrEP (Table 1, Supplemental Table 1). Finally, 94% of providers were comfortable discussing sexual risk behaviors including MSM (ID 100%, non-ID 93%).

**Table 1:**
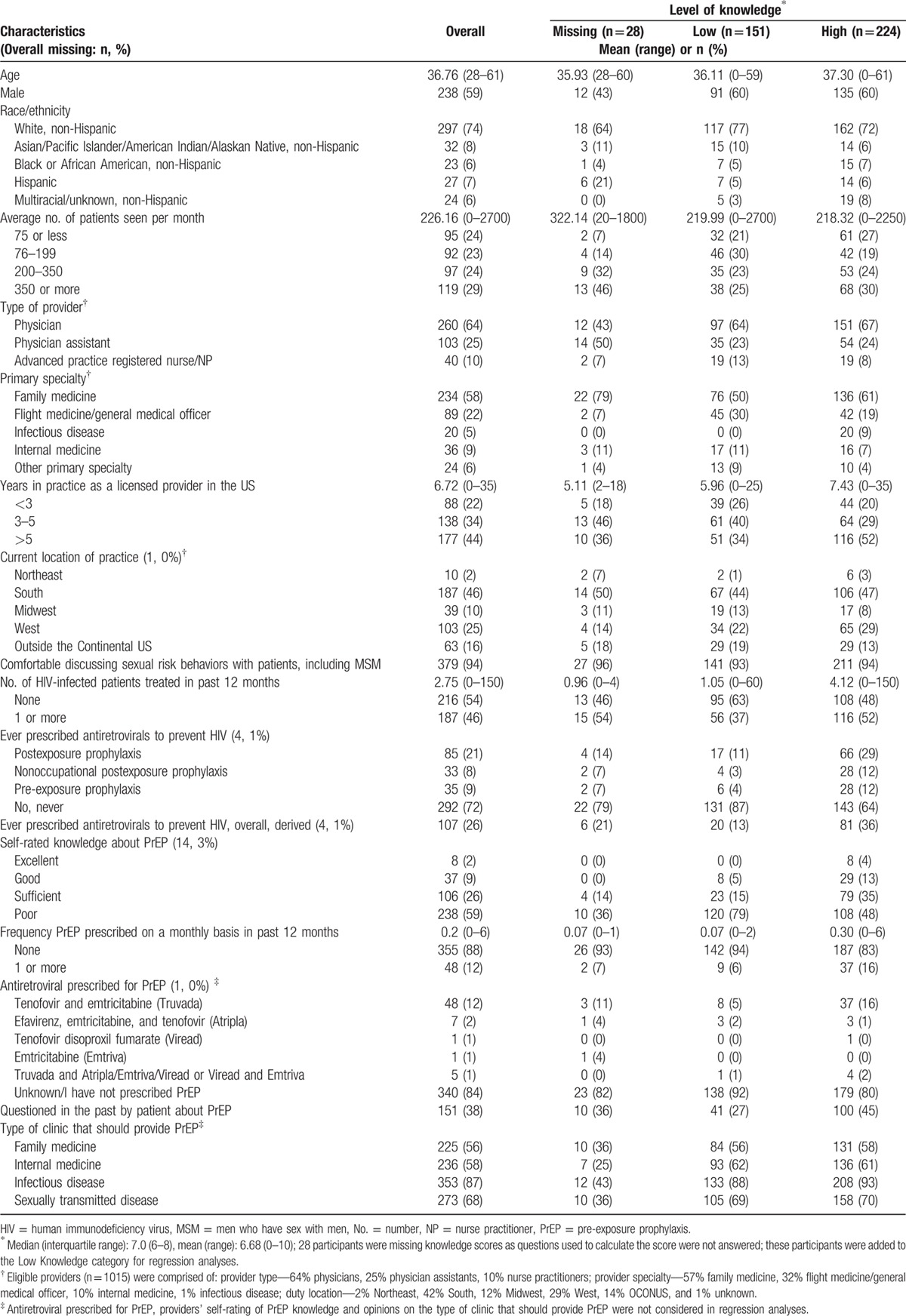
Demographic, medical practice, and HIV pre-exposure prophylaxis (PrEP) experience characteristics of 403 surveyed U.S. Air Force providers, overall and by level of knowledge, December 2015.

### 3.2 Attitudes

**Figure 2:**
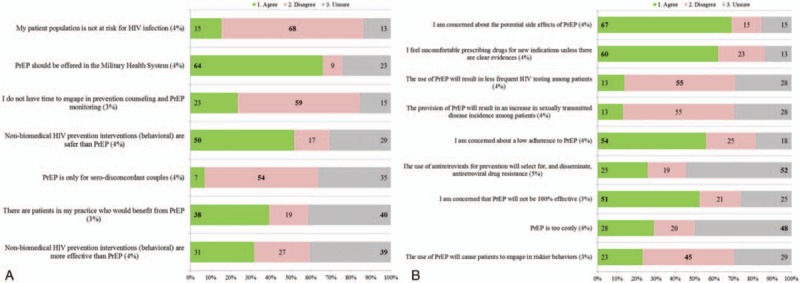
(A) Providers’ beliefs and (B) concerns about HIV pre-exposure prophylaxis. Providers were asked to choose a response (agree, disagree, unsure) to each statement presented on the *Y* axis. The percent in bars reflects frequency of a response by participants. The proportion of participants who did not respond is indicated in parentheses as a percentage. HIV = human immunodeficiency virus.

A majority of participants (overall 64%: ID 85%, non-ID 65%) (Fig. 2A, Supplemental Figure 1A) indicated PrEP should be offered in the Military Health System (MHS) with 87% indicating it should be provided in Infectious Disease clinics and 68% in Sexually Transmitted Disease (STD) clinics (Table 1). Most providers disagreed with the statement that their patient population was not at risk for HIV infection (overall 68%: ID 100%, non-ID 70%) (Fig. 2A, Supplemental Figure 1A) and disagreed that they would not have time for prevention counseling and PrEP monitoring (overall 59%: ID 90%, non-ID 59%).

**Figure 3:**
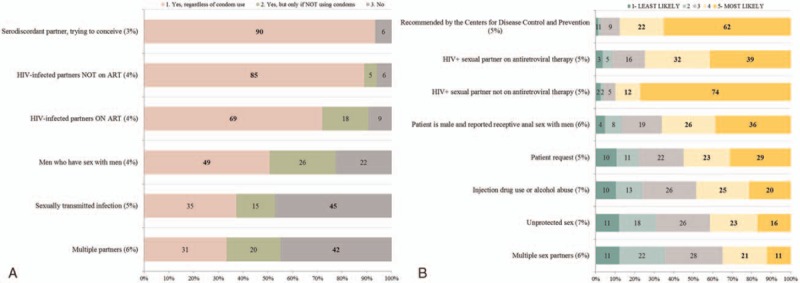
(A) Providers’ beliefs about patient populations who should be offered HIV pre-exposure prophylaxis and (B) primary reasons they would prescribe HIV pre-exposure prophylaxis. For each type of patient population (A) or statement (B) shown on the *Y* axis, providers were asked to respond (A: [1] Yes, regardless of condom use; [2] Yes, but only if NOT using condoms, [3] No; B: scale of 1—Least Likely to 5—Most Likely) whether they would offer PrEP. The percent within bars reflect the frequency of each type of response by participants. The proportion of participants who did not respond is indicated as in parentheses as a percentage. HIV = human immunodeficiency virus, PrEP = pre-exposure prophylaxis.

The main concerns reported by participants included side effects of medication (overall 67%: ID 85%, non-ID 68%, Supplemental Figure 1B) and discomfort with prescribing drugs for new indications without clear evidence (overall 60%: ID 65%, non-ID 62%) (Fig. 2B, Supplemental Figure 2A and B). Other concerns were low adherence by patients (overall 54%: ID 85%, non-ID 55%) and PrEP not being 100% effective (overall 51%: ID 80%, non-ID 51%). Overall 48% of providers, mainly among non-ID respondents (ID 25% vs 51% non-ID), indicated uncertainty about PrEP costs. A large majority of providers thought PrEP should be offered regardless of condom use if a serodiscordant partner was trying to conceive (overall 90%: ID 95%, non-ID 93%) or if an HIV-infected partner was not on ART (overall 85%: ID 90%, non-ID 89%) (Fig. 3A, Supplemental Figure 2). Participants revealed that most frequent reasons they would prescribe PrEP (responses of 4 or 5 on an increasing likelihood scale) were if the CDC recommended it (overall 84%: ID 90%, non-ID 83%) and if a patient had an HIV-infected sexual partner not on ART (overall 86%: ID 100%, non-ID 85%) or on ART (overall 71%: ID 75%, non-ID 71%) (Fig. 3B, Supplemental Figure 3).

### 3.3 Knowledge

Of a maximum score of 10 points, 55% (ID 100%, non-ID 53%) of participants had a high score of 7 points or more. The Cronbach's alpha for internal consistency of the 10 items used for scoring was 0.70. In univariate regression analysis, 8 of 10 demographic, medical practice, and PrEP experience factors were associated with having a high knowledge score (*P* < 0.25) (Table 2). After adjustment for significant factors (*P* < 0.25, Table 2) in a multivariate regression model, 1 factor independently conferred at least a 2-fold higher odds of having a high knowledge score: providers who had ever prescribed antiretrovirals to prevent HIV (AOR: 2.37, 95% CI: 1.27–4.42) (*P* < 0.05) (Table 2).

**Table 2:**
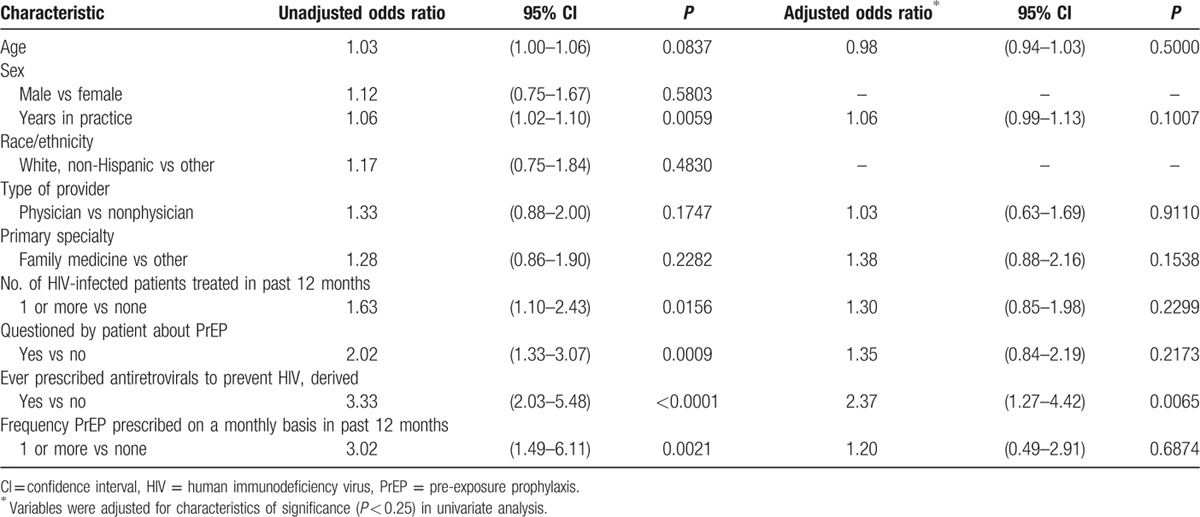
Characteristics of 403 providers associated with a high PrEP knowledge score (n = 224).

## 4 Discussion

We report findings from the first HIV PrEP needs assessment survey conducted only in the Department of Defense (DoD) among 403 active duty USAF physicians, physician assistants, and nurse practitioners practicing in primary care or infectious disease nation-wide or abroad. Although a majority of primary care providers reported never having prescribed PrEP to prevent HIV, most providers thought PrEP should be offered by the military and indicated that their patient population was at risk of HIV infection. The USAF does not have a specific policy regarding PrEP and providers can utilize this strategy at no cost to military beneficiaries at US military bases stateside and overseas. Providers who had prescribed antiretrovirals for PEP or PrEP, scored high on PrEP knowledge questions. However, both PCPs and ID clinicians had concerns regarding medication effectiveness, side effects, and adherence by patients.

More than 3 years after FDA approval of Truvada, only 5% of USAF primary care providers reported ever prescribing PrEP (vs 75% among ID providers), a lower prevalence compared to findings from surveys of non-HIV providers in the US general population.[ ^10^ ^17^] In a survey of providers attending HIV conferences in California and New York who primarily had practiced in Southern California, 15% of non-HIV providers (and 51% of HIV providers) had ever prescribed PrEP. ^[17]^ Similarly, in a 2014–2015 online survey of providers who practiced in 10 US cities with high HIV prevalence, 17% of non-HIV providers (and 61% of HIV providers) had prescribed PrEP. ^[10]^

The low uptake of PrEP by USAF providers may be related to concerns reported in the survey and uncertainty about costs. A majority of PCPs rated their knowledge of PrEP as poor and providers, irrespective of specialty, reported several concerns which included medication side effects (67%), unease in prescribing PrEP without compelling evidence (60%), and low patient adherence (54%). Such concerns have been reported in other surveys ranging from at least 40% of HIV and non-HIV providers reporting concerns of medication toxicity, development of antiretroviral resistance, and lack of patient adherence in 1 US survey to similar major concerns of medication side effects (53%), insufficient evidence of efficacy (53%), cost of PrEP (57%), and antiretroviral resistance and patient adherence (77%) expressed in another survey by ID providers in US and Canada.[ ^10^ ^17^] Training about current practice guidelines and evidence of medication safety and effectiveness of PrEP from recent demonstration projects among MSM, which have shown a reduction of HIV risk by 86% among PrEP users,[ ^23^ ^24^] may be indicated to mitigate providers’ concerns. Lack of knowledge or training has been reported by primary care providers as the main barrier in prescribing PrEP and in providing PrEP education to patients.[ ^10^ ^25^]

Alternatively, the “diffusion of innovation theory” has been offered as an explanation for PrEP uptake in the US in that the initial adoption of PrEP was primarily by innovators and early adopters and not by a majority of prescribers as uncertainty in the early phases of uptake of an intervention is common. ^[26]^ As more major US cities continue to launch PrEP campaigns targeted to the public and health care providers, consequent heightening of awareness may prompt both USAF patients to request PrEP and USAF providers to adopt PrEP as a mainstay in HIV prevention.[ ^27^ ^28^] As an example, implementation of strategic plans for an initiative to end the AIDS epidemic in the state of New York, which included increasing PrEP use among its high-risk HIV-negative population, led to a greater than 5-fold increase in PrEP use among state Medicaid beneficiaries since 2012. ^[29]^ Implementation efforts included the creation of a PrEP toolkit for PCPs, an online PrEP provider directory, a PrEP fact sheet for providers, and an education series on providing health care to MSM. Primary care providers in our survey commonly indicated they most likely would prescribe PrEP if it was recommended by the CDC and did not express time constraints for patient education reported elsewhere. ^[21]^ Moreover, a great majority was comfortable discussing sexual risk behaviors with patients. However, it is unknown what proportions of providers were aware of existing CDC guidelines as this was not a question asked on the survey and may be a gap in knowledge that needs to be addressed. On a related topic of national STD treatment guidelines, 42% of USAF PCPs surveyed from 3 military treatment centers in California indicated they were unaware of CDC guidelines for annual STD screening among MSM. ^[30]^ Furthermore, more than a third reported lack of training in caring for MSM. A recent study in the US shows HIV prevention interventions targeted to high-risk populations, such as PrEP among MSM, is cost effective. ^[31]^ Primary care providers may need to be made aware of such studies and of CDC's clinical practice guideline for PrEP among high-risk populations for reassurance of appropriate utilization of resources in a universal health care delivery system such as the MHS.

Providers, in the majority, indicated their patient population was at risk of HIV infection. However, comparative reporting of being questioned by patients about PrEP was infrequent (less so among patients of ID providers) suggesting either that patient awareness of PrEP or self-perception of sexual risk may be low or that, despite universal health coverage and providers’ indications to the contrary in this survey, patients may be uncomfortable discussing sexual health with their PCP due to service-related barriers or those encountered in the general US population. Almost half (42%) of 1394 MSM surveyed online in 2013 were not comfortable discussing same sex contact with their PCP, 82% had not talked about PrEP with their PCP, and at least 75% believed their PCP would be unwilling to prescribe PrEP. ^[32]^

All ID providers in the USAF, with the exception of 1 provider who was the lead investigator of this survey, participated in this survey and were knowledgeable of, and amenable toward PrEP in the USAF despite having concerns. Although uptake of PrEP in the USAF may be low, and ID providers currently appear capable of meeting demand regarding access to care for PrEP, it is likely that USAF patients’ demand for PrEP may mirror that of the US population's low but escalating use. ^[8]^ Currently in the USAF, PCPs consult with or refer to ID specialists for patients with indications or requests for PrEP. If the rise in PrEP utilization in the USAF starts to mirror national data, PCPs may have to prescribe PrEP without specialty referral, as ID providers are too few in number and geographically dispersed (see Fig. 1) to accommodate this increased need for management and follow up. Alternatively, PCPs may elect to refer patients to civilian ID providers for PrEP which would result in greater cost to the MHS.

## 5 Conclusions

An assessment of PrEP knowledge, perceived risk, and barriers to accessing sexual health services among high-risk USAF patients is merited. In order to optimize PrEP implementation in the US Air Force, courses of action such as training in the form of Continuing Medical Education courses, online webinars or online resources, guidance specific to PrEP in the USAF, and staff dedicated to managing PrEP consults should be considered.

## Supplementary Material

Supplemental Digital Content
