# Supplementary material for: Knowledge, attitudes, and beliefs about HIV pre-exposure prophylaxis among US Air Force Health Care Providers
Source: Medicine (Baltimore). 2016 Aug 12;95(32):e4511. doi: 10.1097/MD.0000000000004511 (PMC4985324; doi:10.1097/MD.0000000000004511)

Supplemental Material

Table 1: Demographic, medical practice and HIV pre-exposure prophylaxis (PrEP) experience characteristics of 403 surveyed U.S. Air Force providers, overall and by provider type (20 infectious disease physicians (ID) and 383 non-infectious disease providers (non-ID)), December 2015

| **Characteristics**  (Missing: n, %) | **Overall**  Mean (range) or n (%) | | **ID** | | **Non-ID** | |
| --- | --- | --- | --- | --- | --- | --- |
| Years in practice as a licensed provider in the US | 6.72 | (0-35) | 9.45 | (0-15) | 6.58 | (0-35) |
| <3 | 88 | (22) | 0 | (0) | 88 | (23) |
| 3-5 | 138 | (34) | 4 | (20) | 134 | (35) |
| >5 | 177 | (44) | 16 | (80) | 161 | (42) |
| Current location of practice (1, 0%) |  |  |  |  |  |  |
| Northeast | 10 | (2) | 0 | (0) | 10 | (3) |
| South | 187 | (46) | 13 | (65) | 174 | (45) |
| Midwest | 39 | (10) | 2 | (10) | 37 | (10) |
| West | 103 | (25) | 3 | (15) | 100 | (26) |
| Outside the Continental US | 63 | (15) | 2 | (10) | 61 | (16) |
| Comfortable discussing sexual risk behaviors with patients, including MSM | 379 | (94) | 20 | (100) | 359 | (93) |
| No. of HIV-infected patients treated in past 12 months | 2.75 | (0-150) | 31.40 | (0-150) | 1.26 | (0-60) |
| None | 216 | (54) | 1 | (5) | 215 | (56) |
| 1 or more | 187 | (46) | 19 | (95) | 168 | (44) |
| Ever prescribed antiretrovirals to prevent HIV (4, 1%) |  |  |  |  |  |  |
| Post-exposure prophylaxis | 85 | (21) | 20 | (100) | 65 | (17) |
| Non-occupational post-exposure prophylaxis | 33 | (8) | 12 | (60) | 21 | (5) |
| Pre-exposure prophylaxis | 35 | (9) | 15 | (75) | 20 | (5) |
| No, never | 292 | (72) | 0 | (0) | 292 | (76) |
| Ever prescribed antiretrovirals to prevent HIV, overall, derived (4, 1%) | 107 | (26) | 20 | (100) | 87 | (23) |
| Self-rated knowledge about PrEP (14, 3%) |  |  |  |  |  |  |
| Excellent | 8 | (2) | 5 | (25) | 3 | (1) |
| Good | 37 | (9) | 11 | (55) | 26 | (7) |
| Sufficient | 106 | (26) | 3 | (15) | 103 | (27) |
| Poor | 238 | (59) | 1 | (5) | 237 | (62) |
| Frequency PrEP prescribed on a monthly basis in past 12 months | 0.2 | (0-6) | 2.00 | (0-6) | 0.10 | (0-3) |
| None | 355 | (88) | 5 | (25) | 350 | (91) |
| 1 or more | 48 | (12) | 15 | (75) | 33 | (9) |
| Antiretroviral prescribed for PrEP (1, 0%) |  |  |  |  |  |  |
| Tenofovir and Emtricitabine (Truvada®) | 48 | (12) | 16 | (80) | 32 | (8) |
| Efavirenz, Emtricitabine, and Tenofovir (Atripla) | 7 | (2) | 0 | (0) | 7 | (2) |
| Tenofovir disoproxil fumarate (Viread) | 1 | (1) | 0 | (0) | 1 | (0) |
| Emtricitabine (Emtriva) | 1 | (1) | 0 | (0) | 1 | (0) |
| Truvada® and Atripla/Emtriva/Viread or Viread and Emtriva | 5 | (1) | 0 | (0) | 5 | (1) |
| Unknown/I have not prescribed PrEP | 340 | (84) | 4 | (20) | 336 | (86) |
| Questioned in the past by patient about PrEP | 151 | (38) | 19 | (95) | 132 | (34) |
| Type of clinic that should provide PrEP |  |  |  |  |  |  |
| Family Medicine | 225 | (56) | 7 | (35) | 218 | (57) |
| Internal Medicine | 236 | (58) | 10 | (50) | 226 | (59) |
| Infectious Disease | 353 | (87) | 19 | (95) | 334 | (87) |
| Sexually Transmitted Disease Clinic | 273 | (67) | 18 | (90) | 255 | (66) |

Figure 1A: Infectious disease physicians (ID, n=20) and non-infectious disease providers’ (non-ID, n=383) beliefs about HIV pre-exposure prophylaxis. Providers were asked to choose a response (agree, disagree, unsure) to each statement presented on the X axis. The percent in bars reflect frequency of a response by participants. The proportion of participants who did not respond is indicated as a percent in parentheses.


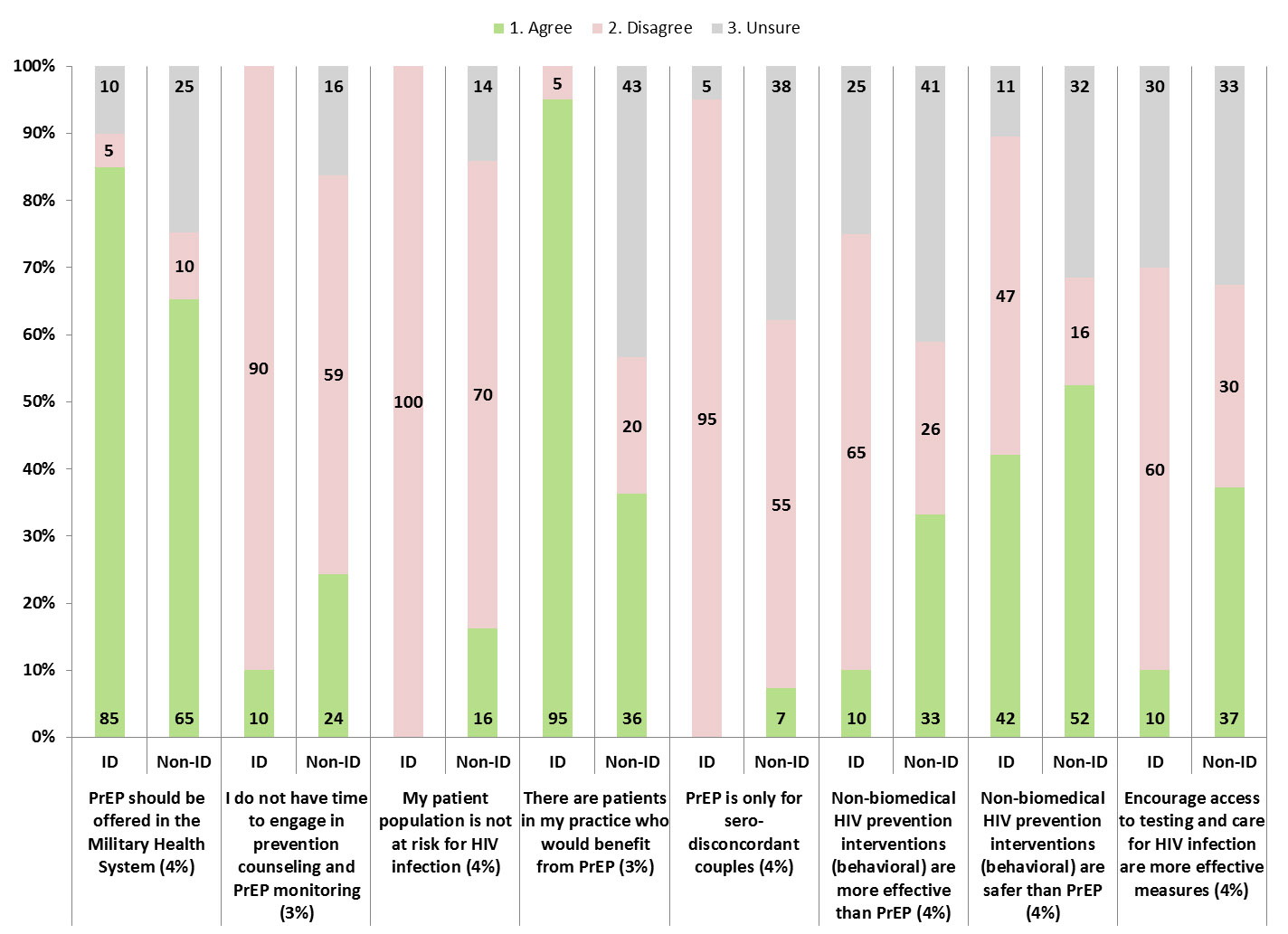


Figure 1B: Infectious disease physicians (ID, n=20) and non-infectious disease providers’ (non-ID, n=383) concerns about HIV pre-exposure prophylaxis. Providers were asked to choose a response (agree, disagree, unsure) to each statement presented on the X axis. The percent in bars reflect frequency of a response by participants. The proportion of participants who did not respond is indicated as a percent in parentheses.


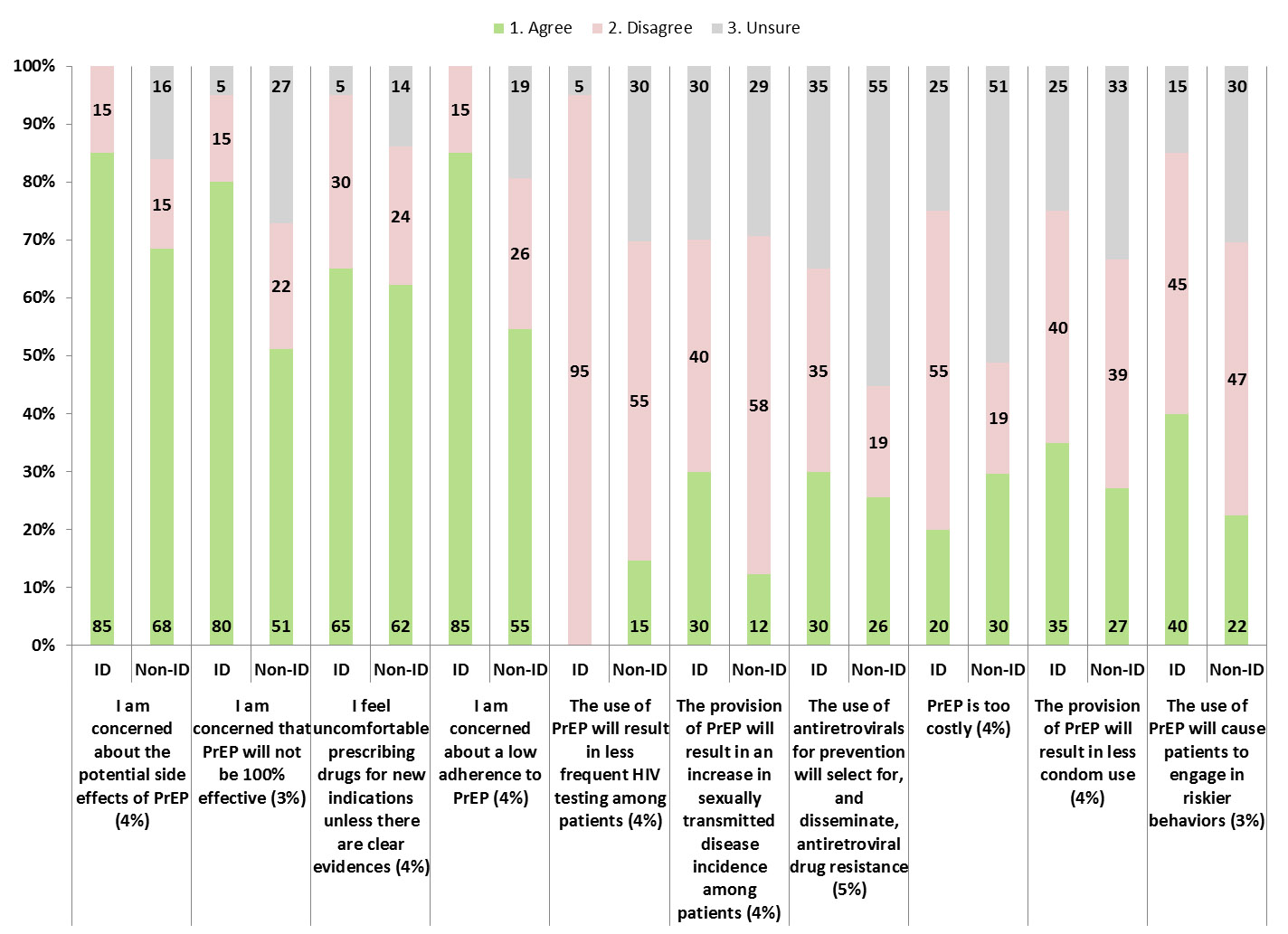


Figure 2: Beliefs about patients who should be offered HIV pre-exposure prophylaxis, by provider type (20 infectious disease physicians (ID) and 383 non-infectious disease providers (non-ID). For each type of patient population shown on the X axis, providers were asked to respond (1. Yes, regardless of condom use, 2. Yes, but only if NOT using condoms, 3. No) whether they would offer PrEP. The percent within bars reflect the frequency of each type of response by participants. The proportion of participants who did not respond is indicated as a percent in parentheses.


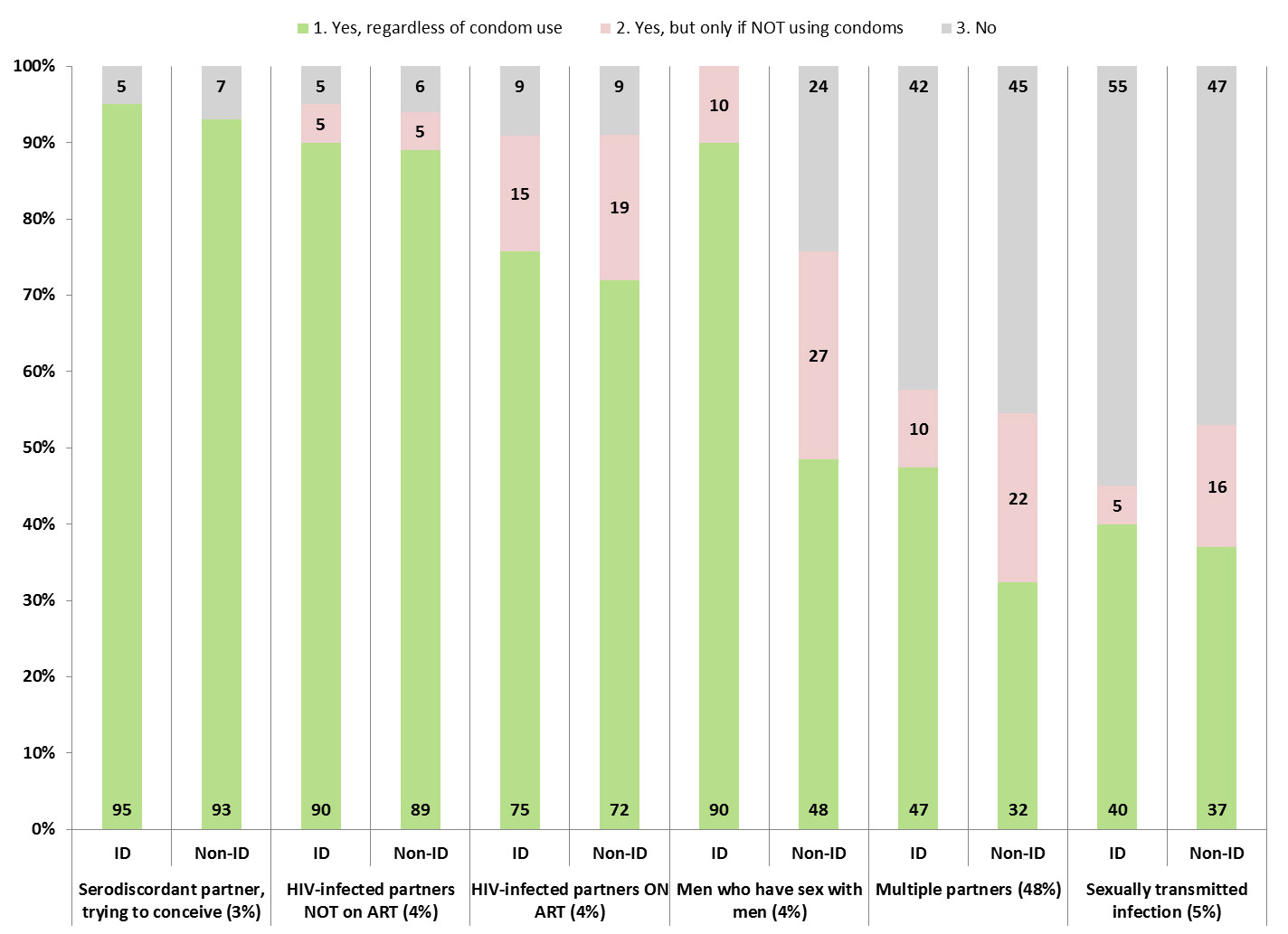


Figure 3: Primary reasons participants believed they would prescribe HIV pre-exposure prophylaxis, by provider type (20 infectious disease physicians (ID) and 383 non-infectious disease providers (non-ID). For each statement shown on the X axis, providers were asked to respond ( from a scale of 1-Least Likely to 5-Most Likely) whether they would offer PrEP. The percent within bars reflect the frequency of each type of response by participants. The proportion of participants who did not respond is indicated as a percent in parentheses.


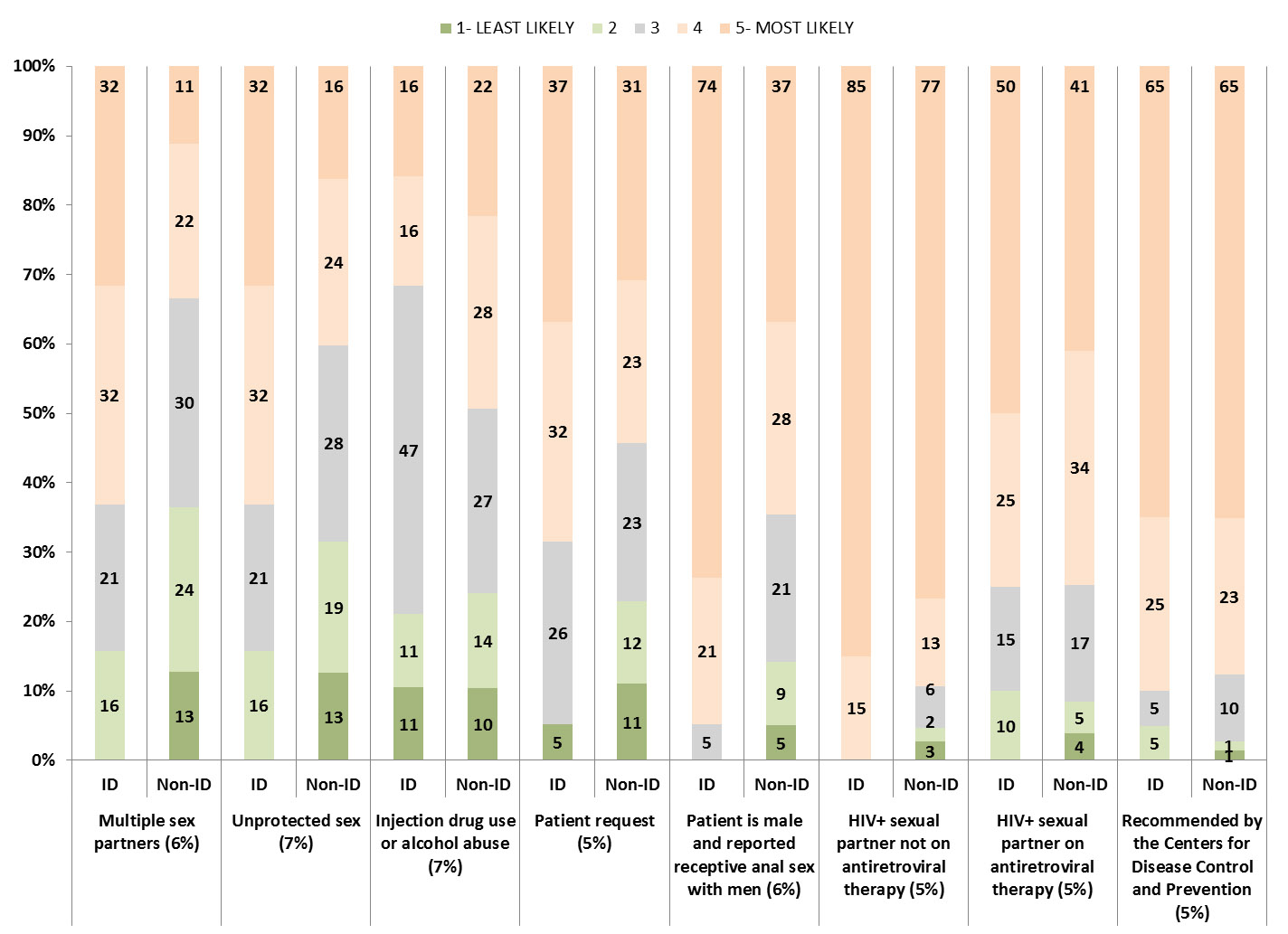

Supplement: Supplemental Digital Content [file medi-95-e4511-s001.doc]
